# Fraud detection and quality assessment of olive oil using ultrasound

**DOI:** 10.1002/fsn3.1980

**Published:** 2020-11-04

**Authors:** Mohammad Reza Zarezadeh, Mohammad Aboonajmi, Mahdi Ghasemi Varnamkhasti

**Affiliations:** ^1^ Department of Agro‐technology College of Aburaihan University of Tehran Tehran Iran; ^2^ Department of Mechanical Engineering of Biosystem Shahrekord University Shahrekord Iran

**Keywords:** adulteration, nondestructive, olive oil, quality, ultrasound

## Abstract

Today, food safety is recognized as one of the most important human priorities, so effective and new policies have been implemented to improve and develop the position of effective laws in the food industry. Extra virgin olive oil (EVOO) has many amazing benefits for human body's health. Due to the nutritional value and high price of EVOO, there is a lot of cheating in it. The ultrasound approach has many advantages in the food studies, and it is fast and nondestructive for quality evaluation. In this study, to fraud detection of EVOO four ultrasonic properties of oil in five levels of adulteration (5%, 10%, 20%, 35%, and 50%) were extracted. The 2 MHz ultrasonic probes were used in the DOI 1,000 STARMANS diagnostic ultrasonic device in a “probe holding mechanism.” The four extracted ultrasonic features include the following: “percentage of amplitude reduction, time of flight (TOF), the difference between the first and second maximum amplitudes of the domain (in the time–amplitude diagram), and the ratio of the first and second maximum of amplitude.” Seven classification algorithms including “Naïve Bayes, support vector machine, gradient boosting classifier, K‐nearest neighbors, artificial neural network, logistic regression, and AdaBoost” were used to classify the preprocessed data. Results showed that the Naïve Bayes algorithm with 90.2% provided the highest accuracy among the others, and the support vector machine and gradient boosting classifier with 88.2% were in the next ranks.

## Graphical abstract

Due to the nutritional value and high price of EVOO, there is a lot of cheating in it. In this study, to fraud detection of EVOO four ultrasonic properties of oil in five levels of adulteration (5%, 10%, 20%, 35%, and 50%) were extracted. The results showed that among the seven different classification models, Naïve Bayes and support vector machine methods and gradient boosting classifier with 90.2%, 88.24%, and 88.24% were the most accurate classification algorithms, respectively.

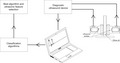

## 1 INTRODUCTION

Fruit of the olive tree (*Olea europaea L*.), which a significant percentage of it, cultivated with the aim of oil extraction, has many nutritional benefits. Consumption of EVOO gets more and more which is a result of people's awareness of its benefits. The original area of the olive is the Mediterranean zone, and its cultivation has several thousand years of old. In general, olive oil is considered as valuable oil due to its high nutritional value, aroma, and taste (Homapour et al., 2016). On average, each olive fruit contains about 15 to 20 percent of the oil. The type and percentage of olive oil ingredients such as fatty acids, antioxidants, and pigments demonstrate its quality, which depends on several factors such as cultivated species, climate conditions, methods of extraction, and ripening stage of the olive fruit. The three factors, which are necessary to be considered in the maintenance of olive oil, are light, time, and temperature. Sunshine to EVOO reduces its quality, so dark‐colored bottle for EVOO is suitable. In today's world, food safety and health are recognized as one of the most important human priorities, so new policies have been legislated to ensure quality assurance and food security to improve and develop effective laws in the food industry which support the control of food chain from farm to fork, and it provides the capacity for intelligent monitoring systems to human health and consumer confidence security. Contaminants threatening food safety and health can be microbial, foreign bodies (include biological, chemical, or physical), natural toxins, and other chemical compounds and packaging materials (West et al., 2008). Although food chain contaminants can be detected by laboratory methods, bioengineers are looking for technologies to detect contaminated food faster, easier, and more effectively. Laboratory biological tests to detect food contaminants are time‐consuming, complex, and sometimes lack precision, so it is necessary to attend pigment to new diagnostic technologies in food industries.

The use of ultrasonic technologies in the food industry is becoming popular these days. These applications include drying, homogenization, emulsification, oil extracting, pigment extraction, food processing, elimination of microorganisms, fraud detection, quality evaluation, and detection of agricultural product ripening. These cases are studied in two general categories: diagnostic ultrasound and power ultrasound. The diagnostic ultrasound in foodstuffs is based on the difference in signal velocity, attenuation coefficient, etc., which varies in different materials. There are two types of diagnostic ultrasounds to determine the quality of liquid and agro‐food products: pulse‐echo (just one sensor) and pulser‐receiver (two sensors on both sides on the sample). Changing the transmitter signal properties, after passing through the sample, will be base for evaluating. Ultrasonic signal's velocity is very sensitive to molecular structure and intermolecular connections, which is suitable for determining the composition, structure and physical state, and detecting a foreign body and defect in processed and packaged foods. The denser material has a faster velocity of the ultrasonic wave (Bernat‐Maso et al., 2017; Laugier & Haïat, 2011; Meftah & Mohd Azimin, 2012). Ultrasonic energy decreases by increasing the distance from the source of the signal which is a result of signal amplitude. In addition to velocity, attenuation coefficient, and acoustic impedance, other parameters rely on the sample material. When the ultrasonic wave passes thorough in a medium, it loses its energy gradually, which is expressed as attenuation. The attenuation coefficient (*α*) is obtained according to Equation (1) (Rao et al., 2014):

(1) $$A=A_{0}e^{‐\alpha x}$$

where “*x*” is the distance that the wave has travelled in the medium, “*A*_0_” and “*A*” are the amplitude of the reflected wave at the beginning of the wave and after passing “*x*” distance, respectively. The attenuation coefficient increases with decreasing temperature and increasing frequency (McClements & Povey, 1992).

**FIGURE 1:**
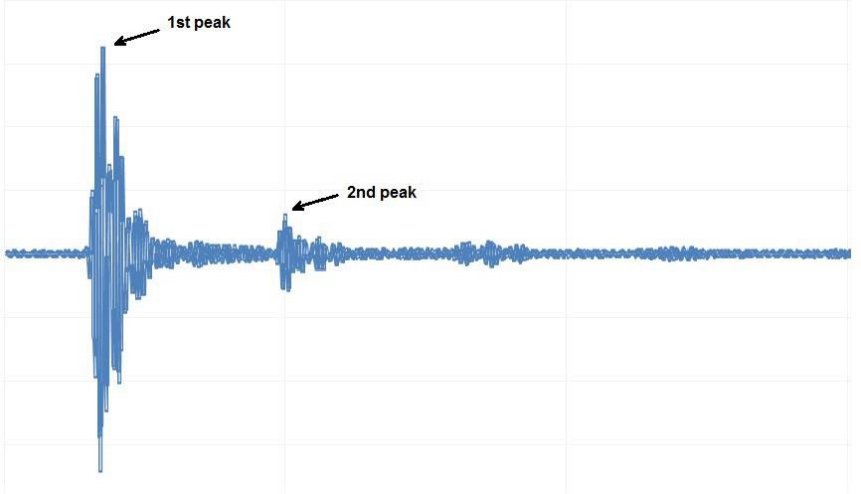
Schematic of signal received by receiver probe

According to Figure 1, in each diagnostic ultrasound test, there is more than one peak for amplitude in the time‐domain diagram, which is a result of wave reflections.

In diagnostic ultrasonic systems, it is necessary to note that propagation of ultrasonic waves from the air is difficult; the presence of air in the path of the waves leads to the attenuation of the wave. Of course, in air‐coupled ultrasonic systems, this is not a serious problem (Fathizadeh & Aboonajmi, 2017). Usually, there is an air gap between ultrasonic probe and the test sample's container, so it is necessary to use a material for coupling. The matter which uses as a coupler can be oil (Meftah & Mohd Azimin, 2012), water (Hæggström & Luukkala, 2001; Zhao et al., 2003) and so on. Also in diagnostic ultrasonic tests, it is important to keep the temperature of the test sample constantly, because the ultrasonic properties vary with temperature, and ignoring these changes can lead to errors in results. If the temperature changes during the test, the results should be modified by applying the coefficients. According to Equation (2), as the temperature decreases, the speed of the ultrasonic wave increases, and vice versa. Increasing the frequency of ultrasonic waves will also lead to an increase in the wave velocity (McClements & Povey, 1992).

(2) $$V=3.3\;m\;s^{‐1}\;{}^{\circ}C^{‐1}$$

Diagnostic ultrasound has been widely used in food quality assessment, agriculture, livestock, fisheries, etc. This approach has been used in various researches, including detection of fruit ripening and harvest time (Mizrach, 2008; Morrison & Abeyratne, 2014), the presence of foreign bodies in packaged foods (Zhao et al., 2003), and determination of egg quality (Aboonajmi et al., 2010). Research on fruit quality assessment may be performed for sliced fruit (destructive) or for whole fruit (nondestructive). In any way, the basic of the test is the same in both methods and is based on changes in ultrasonic properties such as velocity and wave attenuation. Some of the studies on food quality assessment by ultrasonic technology are given in Table 1.

**TABLE 1:** Some research on diagnostic ultrasound in food quality evaluation

Sample	Mode	Frequency	Reference
Vegetable oils	Pulser‐Receiver	1 MHz	Rashed, & Felfoldi (2018)
Coconut oil	Pulse‐Echo	1 MHz	George et al. (2017)
Some liquid mixtures	Pulse‐Echo	100 Hz	Cooke (2016)
Olive oil	Pulse‐Echo	2.25 MHz	Alouache et al. (2015)
Oranges	Pulse‐Echo	100 kHz	Morrison & Abeyratne, (2014)
Frying oils (USBO, PHSBO)	Pulser‐Receiver	5 MHz	Izbaim et al. (2010)

The most common approach for evaluation of olive oil quality which is performed in food laboratories is the chromatography method which is a destructive test. Generally, chromatography is in two types: gas chromatography (GC) and high‐performance liquid chromatography (HPLC). Chromatography is based on the separation of the sample (in this study: olive oil) into the components. Also, there are some other methods for food quality assessment such as olfaction machine (Xu et al., 2016), calorimetry (Van Wetten et al., 2015), machine vision (Jafari et al., 2014), spectroscopy (Ok, 2017), and many other methods.

There are several methods for analyzing and classification of data such as artificial neural network (ANN), gradient boosting classifier (GBC), support vector machine (SVM), K‐nearest neighbors (KNN), blockchain, and many other models. These models have a wide application in food quality evaluation and classification (Galvez et al., 2018; Gonzalez‐Fernandez et al., 2019; Liu et al., 2019; Martinez‐Castillo et al., 2019; Pan et al., 2019; Qiu & Wang, 2017).

With respect to the fact that most of the well‐established methods of olive oil quality assessment, such as GC, are nondestructive and slow, the presented method is nondestructive, fast, and easy to use. Ultrasound parameters of each matter are unique and known as a fingerprint of matter, so in this study, the diagnostic ultrasonic method for olive oil quality evaluation was used. Also in this study, several different classification models were used for analysis and classification, so determining the best classification method is other aims of the study. ,lfjb

## 2 MATERIALS AND METHODS

**FIGURE 2:**
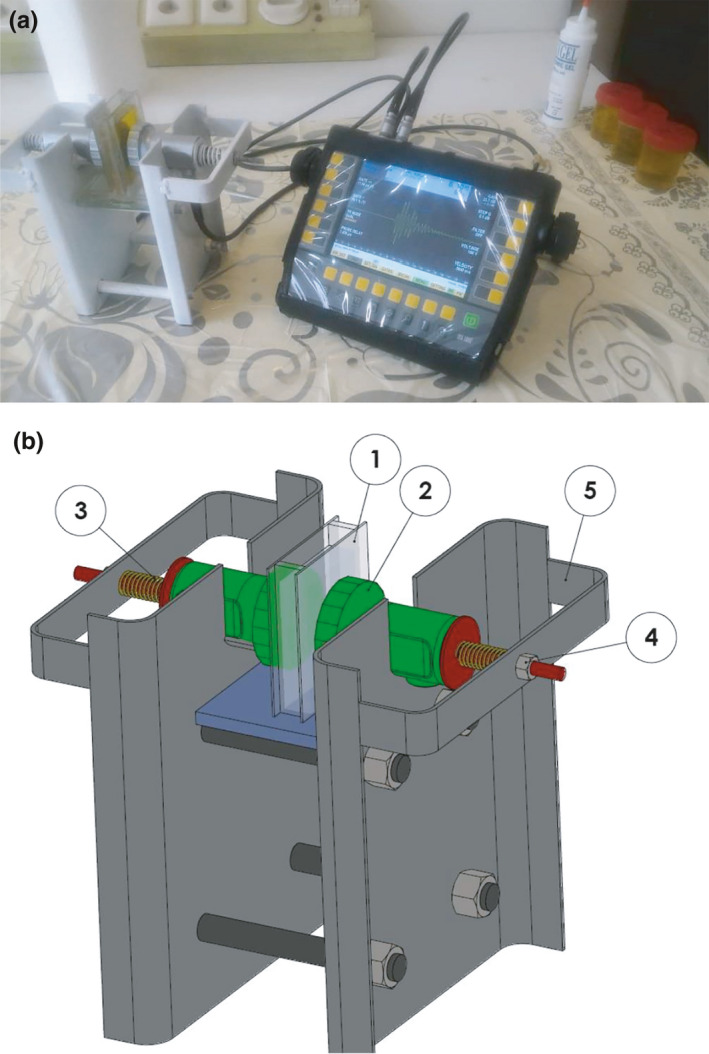
(a) Used diagnostic ultrasound device, (b) structure of probes holder (1. sample (oil), 2. ultrasonic probe (2MHz), 3. compression spring, 4. adjusting nut, and 5. main body)

In this study, a diagnostic ultrasonic system was used to receive the ultrasonic properties of various samples of extra virgin olive oil and its blends with different percentages (by common frying oil on the market). According to Figure 2, this system consists of the DIO 1,000 STARMANS diagnostic ultrasonic device, a couple of 2 MHz transmitter and receiver probe (Model 57745, Series B2S), the guide of probes system, and the oil sample chamber which is made up of thin glass (1 mm thickness). The sample chamber was coupled with probes by ultrasonic.

### 2.1 Preparation of samples

The EVOOs used in this study were obtained from a well‐known factory in a town named Lowshan in the North of Iran. Oils in this study were extracted by cold press method. Also, fatty acid profiles were determined in a food laboratory by “Agilent Technologies (Model 7890B)” gas chromatography system. Gas chromatography (GC) is a method for detecting and separating volatiles of a substance, usually liquid or gas. The EVOOs were mixed with common frying oil including sunflower oil, canola oil, and corn oil sold in markets to make 6 classes for fraud creation of 5, 10, 20, 35, and 50 percentages in mass.

Out of each class of frauds, four samples were prepared with 100 ‐gram net weight and tests for each sample were repeated seven times, so a totally of 28 tests were done for each class. As mentioned above, three important factors that affect the quality of olive oil are light, temperature, and oxygen. Therefore, the samples were kept in a dark place and at lower than 25°C temperature. To minimize oxidation of samples, it is necessary to minimize the amount of oxygen in the sample container, so sample containers were filled completely. The profile of fatty acids has been shown in Table 2.

**TABLE 2:** The sample's profile of fatty acid

Type	C16:0	C17:0	C17:1	C18:0	C18:1c	C18:2c	C18:2t	C18:3	C20:0	C22:0	C24:0
EVOO	15.72	0.03	0.06	2.48	66.05	12.59	0.02	0.64	0.44	0.12	0.08
EVOO with 5% Adulteration	15.22	0.04	0.07	2.54	63.75	14.35	0.03	0.8	0.42	0.14	0.09
EVOO with 10% Adulteration	14.76	0.03	0.01	2.56	63.1	16.24	0.03	0.8	0.42	0.17	0.1
EVOO with 20% Adulteration	13.8	0.04	0.06	2.56	60.8	19.41	0.03	0.93	0.41	0.22	0.11
EVOO with 35% Adulteration	12.38	0.04	0.06	2.63	57.44	24.04	0.04	1.09	0.4	0.3	0.013
EVOO with 50% Adulteration	11.7	–	0.06	2.72	55.45	26.52	0.05	1.2	0.4	0.35	0.16
Common frying oil	5.87	0.03	0.05	3.03	40.63	46.1	0.07	1.98	0.34	0.68	0.27

The results of chromatography experiments on the samples showed that the percentage of some fatty acids such as oleic acid (C18:1) and palmitic acid (C16:0) decreased by increasing the percentage of EVOO in the blend, which was not unexpected.

### 2.2 The guide of probe system

**FIGURE 3:**
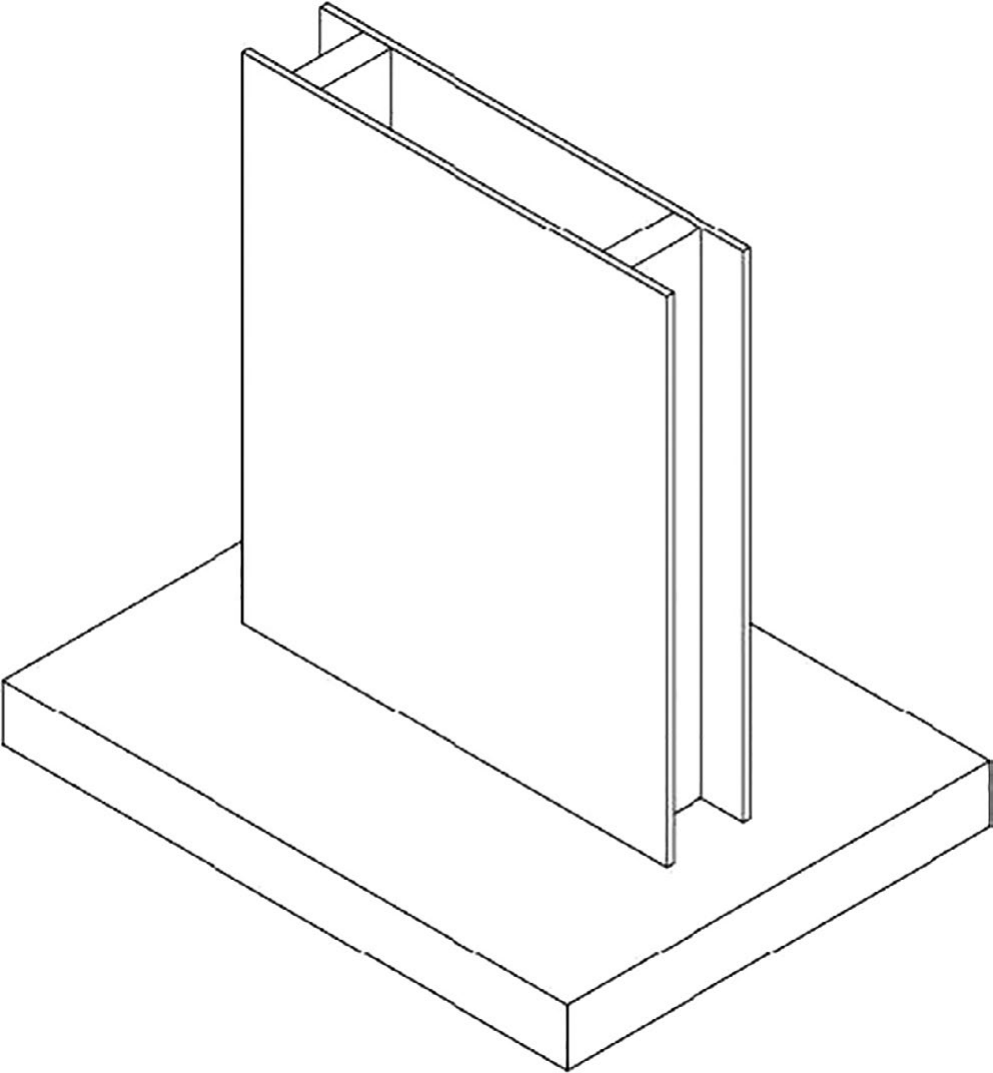
Sample chamber of oils

A thin (one millimeter) glass was used to make the oil sample container. According to the dimensions of the transmitter and receiver probes of the ultrasonic system (the outer diameter of the probe in the contact zone with the sample container was about 30 mm), a small container for storing sample oil between the two probes was designed and manufactured according to Figure 3. According to item 1 in Figure 2b, the distance between the transmitter and receiver probes was 12 mm, and after decreasing the thickness of the glass, the wave travels just ten millimeters in the oil sample. Ultrasonic systems are very sensitive to the pressure applied on the probes coupling with the sample chamber, so the guide of probes system was designed using SOLIDWORKS 2016 software. Pretests have been done on the mentioned prototype of the device, and then, the final model was manufactured and optimized. The adjustable pressure on the probes for coupling (with the help of adjusting screws) made this system possible for all tests to have the same conditions. Coupling the probes to the sample chamber has been done by ultrasound lubricant gel.

Figure 1 shows an example of system output. The raw data were transferred to a computer via a USB port and converted into processable data by MATLAB R2014a software. Four features of the ultrasonic signals were extracted: 1. percentage of amplitude reduction from the transmitter to the receiver (which is an index of signal attenuation) 2. time of flight (TOF), 3. amplitude reduction from the first peak to the second peak in the time–amplitude diagram (Figure 1), and 4. the ratio of the first peak to the second peak of amplitude.

To know the data dispersion, all the data (four features of ultrasound properties) were analyzed by a box plot and a histogram.

(3) $$Z=\frac{\left(x‐\mu \right)}{s}$$

**FIGURE 4:**
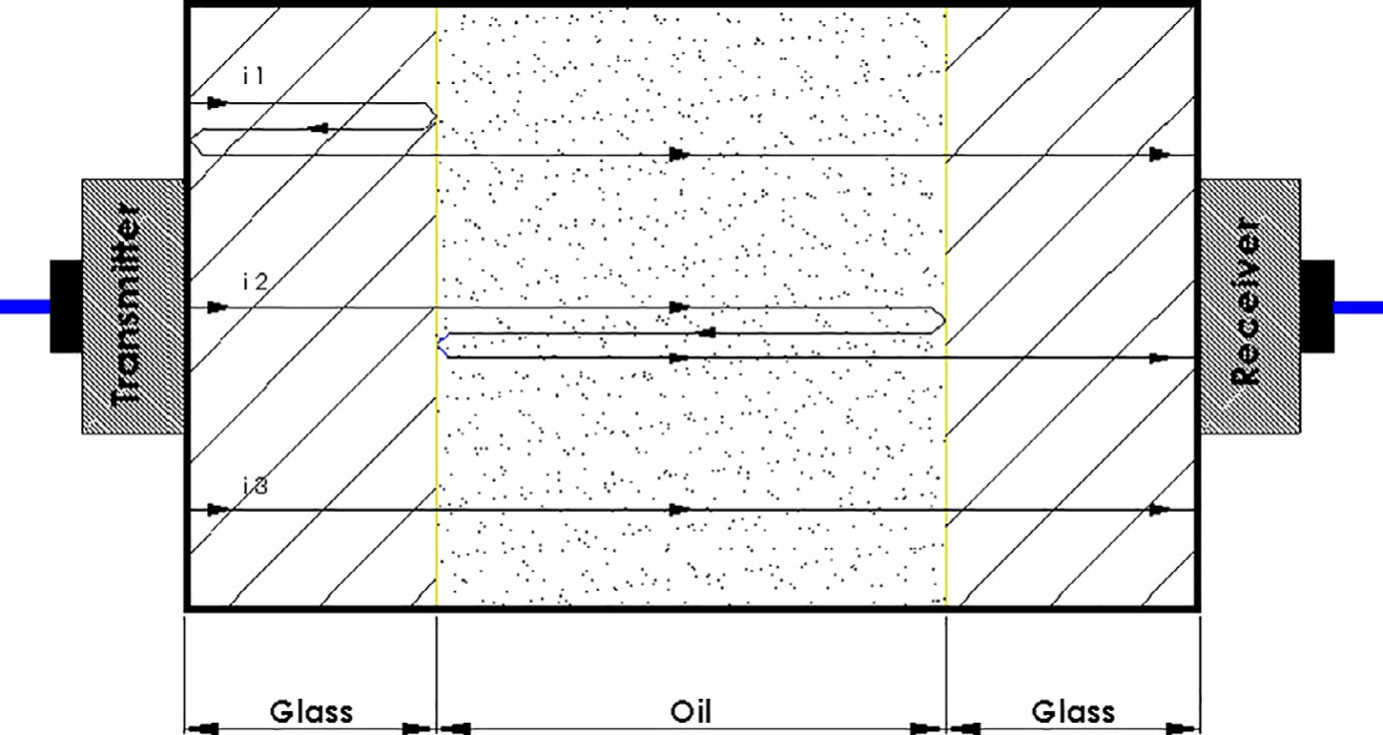
Reflections of signal when changes the medium

Ultrasonic waves continue to move directly or indirectly as they pass from one material to another, or reflect or refract with a certain angle. So, in this study, as shown in Figure 4, because the wave path includes several material, so we have a few wave reflections and frequently peak in the time–amplitude diagram. Wave reflection has been taken place when the ultrasonic wave travels from a matter to the other (that includes wave traveling from transmitter probe to glass, from glass to oil, oil to glass, and glass to receiver probe).

Combining low‐value, low‐price oils with extra virgin olive oils changes physical properties such as density and homogeneity, and has a direct effect on velocity, attenuation, and refraction coefficient. A diagnostic ultrasonic system combined with a machine learning system can be easily used to detect various purities of extra virgin olive.

In this study, preprocessing codes, as well as data classification operations, were written and run using Python software version 3.7.

## 3 RESULTS AND DISCUSSION

**FIGURE 5:**
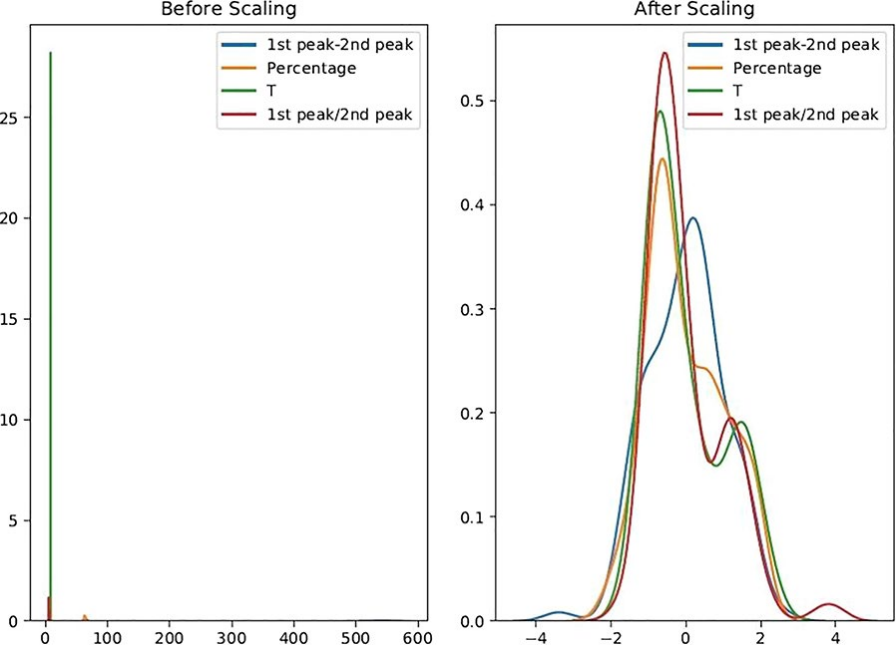
Effect of normalization on status of data distribution

Basically, before modeling the data with most of the classification algorithms (such as neural networks, SVM, and KNN), it is necessary to normalize the data; raw data lead to an unbalance effect of each feature and it is undesirable. Figure 5 shows the effect of normalization on data distribution. Normalization will be very important when features and data are in different scales. For evaluation of the data dispersion, all the data (four ultrasound features) were analyzed by a box plot and a histogram. These diagrams showed that the outliers in the data are considerable. Also, charts indicate that the distribution of the data is more than normal value and need to preprocessing operation. Preprocessing will improve the accuracy of results, and sometimes without preprocessing, analyzing the data is impossible.

In statistical multivariate analysis, there are different methods for measuring dependence or relationship between two random variables. The correlation between two variables means the prediction of the value of one parameter by another.

Table 3 shows the correlation matrix between the features. Correlation matrix values are in the range of −1 (maximum reverse correlation) to the + 1 (maximum correlation). The zero correlation coefficient indicates that the two parameters are not interdependent.

**TABLE 3:** Correlation matrix between the features

	Amplitude loss	TOF	1st peak−2nd peak	1st peak/2nd peak
Amplitude loss	1	−0.37	0.73	−0.42
TOF	−0.37	1	−0.01	0.56
1st peak−2nd peak	0.73	−0.01	1	0.28
1st peak/2nd peak	−0.42	0.56	0.28	1

**FIGURE 6:**
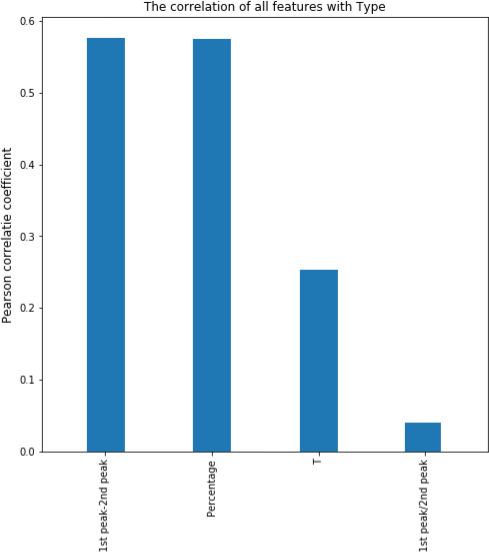
Relationship between extracted features with type

Also, the relationship between ultrasonic properties and the desired class (correlation coefficient) was compared (Figure 6). According to this diagram, the “amplitude reduction from the first peak to the second peak in the time–amplitude diagram” and the “percentage of amplitude reduction from the transmitter to the receiver” showed the highest correlation with the class.

According to Table 4, the “amplitude reduction from the first peak to the second peak in time–amplitude diagram” with 32.22% was the most important feature. Attenuation coefficient of oil obtains from this feature. The “the ratio of the first peak to the second peak of amplitude” with 18.15% showed the least effect on classification.

**TABLE 4:** Extracted ultrasonic features sorted by importance

Features	Percentage
1st peak–2nd peak	32.22
Reduction of amplitude (%)	29.13
Time of flight	20.50
1st peak/2nd peak	18.15

**FIGURE 7:**
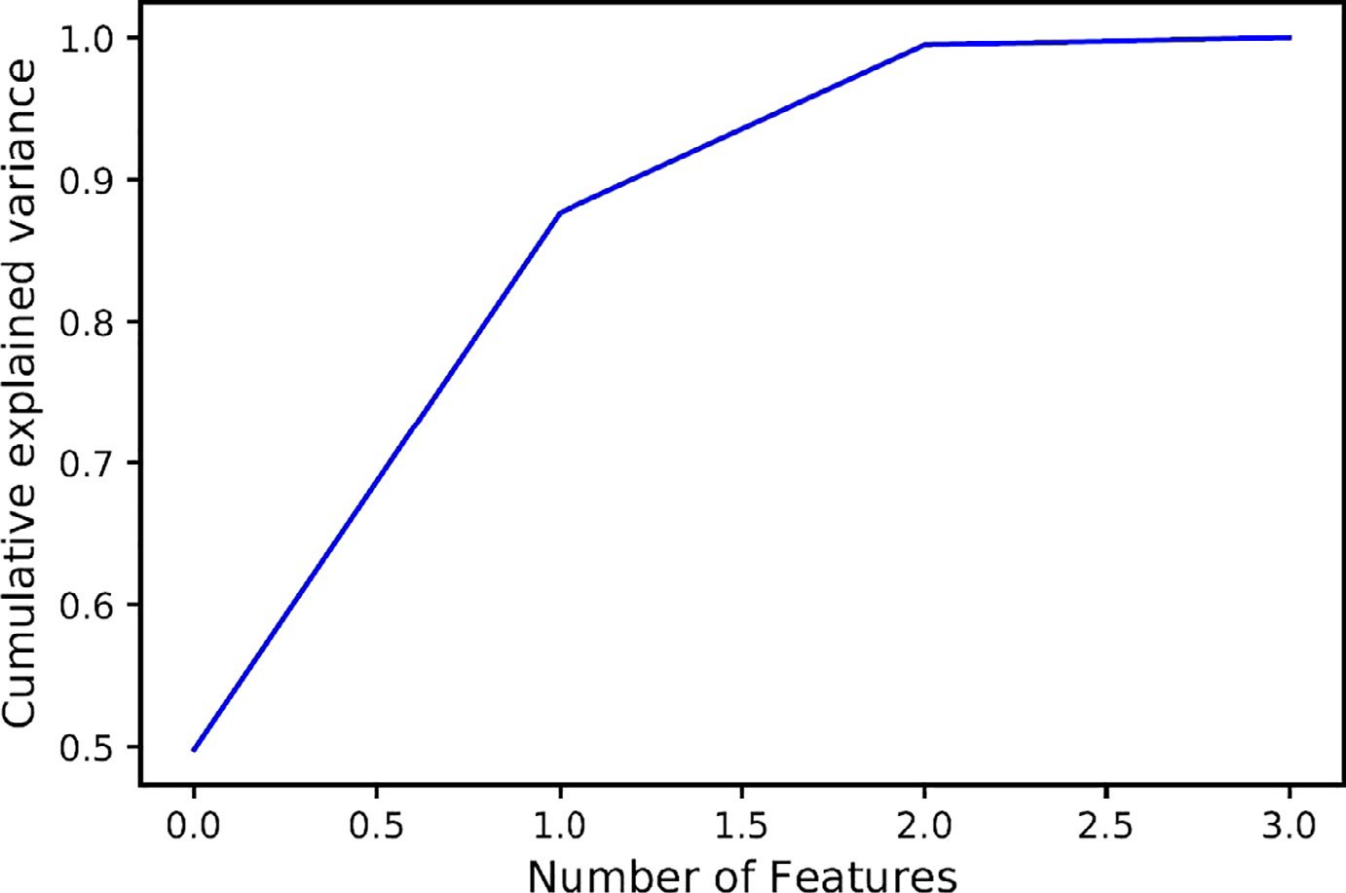
Principle component analysis

Figure 7 shows the principal component analysis (PCA) for data. According to this figure, with the elimination of one of the four ultrasonic properties, there is no noticeable change in the accuracy of the classification, and just with three features, high accuracy of classification (96%) can be obtained. The PCA method is one of the most important results of linear algebra. In this method, data are moved from a high‐dimensional space to a low‐dimensional space. PCA is a feature selection method that is used in dimension reduction applications; analyzing in lower dimension space is easier and faster.

**FIGURE 8:**
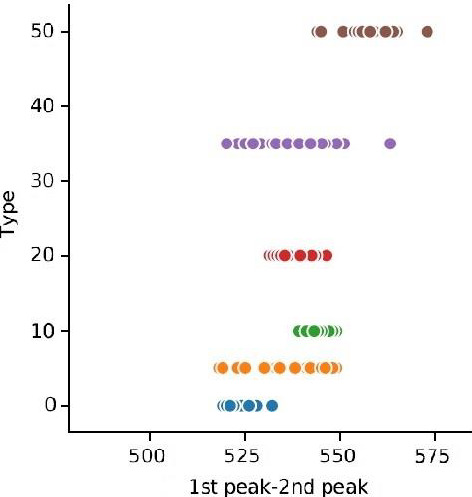
Treatment of the “difference between the first and second peak of amplitude” feature with changing in fraud

The trend of “the difference between the first and second maximum amplitudes of the domain” with changes in oil adulteration is given in Figure 8. It is observed that an increase in the percentage of fraud oil to the sample leads to an increase in the difference between the values of the first two peaks; in other words, increasing in the percentage of fraud oil leads to decrease in amplitude and increase in signal attenuation.

### 3.1 Classification algorithm

For validation of classification algorithms, it is necessary to divide the data into two classes of “train data” and “test data.” This requires sufficient amount of data. If the number of data is less, the results will not be valid.

In this study, 70% of data assigned to train data and 30% to test data. These percentages were determined empirically. In this study, the Naïve Bayes classification algorithm with 90.20% was the best model in classification accuracy. Naïve Bayes is a simple and well‐known classifier that provides good results when fewer data are available. These methods are a simple probability classifier that calculates a set of probability by counting the frequency and combinations of values in a given data set. The confusion matrix of the Naive Bayes method for EVOO classification is presented in table 5.

**TABLE 5:** Confusion matrix of Naïve Bayes method

		Predicted					
		0%	5%	10%	20%	35%	50%
Actual	0%	10	0	0	0	0	0
	5%	1	6	0	0	1	0
	10%	0	0	8	0	0	0
	20%	0	0	1	9	0	0
	35%	1	2	0	0	5	0
	50%	0	0	0	0	0	7

This table shows that this method can detect fraud samples with high accuracy. After the Naïve Bayes method, the SVM and gradient boosting classifiers were evaluated both with 88.24% accuracy.

Also, AdaBoost and logistic regression classification models had the lowest classification accuracy compared to other models (52.94% and 74.51%, respectively). These methods may show a higher accuracy if the quantity of data was more.

**FIGURE 9:**
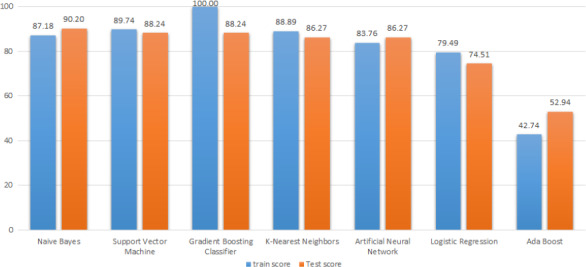
Classification results with 7 different models

The results of the data classification with the models mentioned above are given in Figure 9.

## 4 CONCLUSION

The results showed that among the seven different classification models, Naïve Bayes and support vector machine methods and gradient boosting classifier with 90.2, 88.24, and 88.24%, were the most accurate classification algorithms, respectively. AdaBoost method also provided the lowest accuracy with 52.94%. Among four extracted ultrasonic features “1. percentage of amplitude reduction, 2. time of flight (TOF), 3. the difference between the first and second maximum amplitudes of the domain (in the time–amplitude diagram), and 4. the ratio of the first and second maximum of amplitude,” the 3rd feature showed the greatest effect on the accuracy of data mining. The results showed that by extracting various ultrasonic properties and learning those data to the machine, the fraud of 5% in the extra virgin olive oil can be detected with high accuracy.
